# Thermomechanical Fractional Model of TEMHD Rotational Flow

**DOI:** 10.1371/journal.pone.0168530

**Published:** 2017-01-03

**Authors:** F. Hamza, A. Abd El-Latief, W. Khatan

**Affiliations:** 1 Department of Mathematics, Faculty of Science, University of Alexandria, Alexandria, Egypt; 2 Department of Mathematics, Faculty of Science, University of Damanhour, Damanhour, Egypt; Massachusetts Institute of Technology, UNITED STATES

## Abstract

In this work, the fractional mathematical model of an unsteady rotational flow of Xanthan gum (XG) between two cylinders in the presence of a transverse magnetic field has been studied. This model consists of two fractional parameters *α* and *β* representing thermomechanical effects. The Laplace transform is used to obtain the numerical solutions. The fractional parameter influence has been discussed graphically for the functions field distribution (temperature, velocity, stress and electric current distributions). The relationship between the rotation of both cylinders and the fractional parameters has been discussed on the functions field distribution for small and large values of time.

## Introduction

In many engineering fields such as electrical, mechanical, and nuclear engineering, the study of the fluid flow coupled with heat transfer in rotating annuli has great importance in applications [1]. The rotating flow is applied in several industrial applications such as turbo-machines, thermal motors and especially in turbines. The inner rotating flow is used in revolving jets and devices of combustion in order to increase the mixture between the reagents, as well as to stabilize the flame or to obtain advantages of better mixing [2]. A non-Newtonian fluid has many models that are applied in order to discuss the behavior of different materials; such as, drilling mud, certain oils, greases, and blood [3]–[5]. The oil industry has a great attention to the flow of fluids in annular spaces, both in drilling, and the artificial lifting of oil [6]. The second grade fluids are the common non-Newtonian viscoelastic fluids in industrial fields, such as polymer solutions [7, 8]. Laplace transform has widely used to obtain the exact solution of unsteady Magneto-hydro-dynamics (MHD) for different types of flow [9]–[17]. Thermo-electric magneto-hydro-dynamics (TEMHD) theory was originally developed by Shercliff for direct application in a fusion environment [18]. The thermoelectric effect develops the current between a liquid metal and a container wall when there is a temperature gradient along the interface between them [19, 20].

Recently, fractional calculus has encountered a great success in the description of viscoelasticity [21]–[23]. This was achieved by replacing the time derivative of an integer order by its fractional one. This process now allows one to define precisely the non-integer order derivatives. The Generalized second-order fluid with fractional anomalous diffusion studied by Xu et al. [24], while the fractional derivative to the constitutive relationship models of Maxwell viscoelastic fluid and second grade fluid had been studied by Wenchang et al. [25, 26]. Fractional Maxwell fluid was examined for unsteady Couette flow by Athar et al. [27]. The oscillating flows in a generalized second grade fluid was studied by Jamil et al. [28]. The second grade fluid flow between two cylinders was studied differently, considering the flow of such fluid [29, 30]. Sherief et al. derived the fractional order theory of thermoelasticity by using fractional calculus [31]. Sherief and Abd El Latief have solved 1D problems [32, 33], and 2D [34] in the context of this modified fractional theory. They also studied the effect of the fractional derivative parameter on fractional thermoelastic material with variable thermal conductivity [35]. Abd El-Latief and Khader applied this theory to a 1D problem for a half-space overlaid by a thick layer of a different materials [36]. Ezzat [37, 38] constructed a mathematical model of the TEMHD in the context of the fractional heat conduction equation by using the Taylor series expansion of time fractional order developed by Jumarie [39]. Hamza et al. [40] modified the generalized theory of thermoelasticity with two relaxation times to be of fractional order derivative.

Here, we will introduce a fractional thermomechanical model with two parameters *α* and *β* for an unsteady rotational flow of thermoelectric fluids in the presence of a transverse magnetic field. Numerical solutions are obtained in the Laplace transform domain. The solutions in the physical domain are obtained numerically by using the Laplace inversion process based on a Fourier-series expansion. The Numerical results for the functions field distribution are represented graphically for different values of *α* and *β* with small and large values of time *t*. These graphs are analyzed for the counter direction rotation of two cylinders from which we can observe the physical behavior of these thermomechanical fractional parameters.

## Formulation of the problem

**Fig 1:**
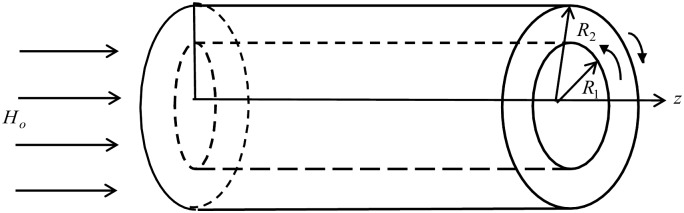
The geometry of the problem.

Consider an unsteady laminar flow of an incompressible thermoelectric generalized second grade viscoelastic fluid situated in the annular region between two infinite coaxial isolated circular cylinders of radii *R*_1_ and *R*_2_ (*R*_2_ > *R*_1_). Then the cylinders suddenly begin to rotate, about their common axis *r* = 0 in different directions with a thermal shock that is a function of time. The surfaces of the cylinders are taken to be traction free where a constant magnetic field of strength *H*_0_ acts axially in *z*-direction. The magnetic Reynolds number is assumed to be so small that the induced magnetic field is neglected. Due to the formulation of the problem with cylindrical coordinates (*r*, *ϕ*, *z*), all variables field depend on *r* and *t* only in *ϕ*-direction; as illustrated in Fig 1.

To clarify the physical applications of the graph, the assumptions [41] are required in cylindrical coordinate as follow:

1. The fluid between the cylinders moves gradually with velocity ***V*** = (0, *v*(*r*, *t*), 0) while the constitutive equation of generalized second grade fluid, corresponding to this motion is given by [7]
  $$\begin{array}{c} \tau \left(r,t\right)=\left(\mu +\alpha_{1}D_{t}^{\beta }\right)\left(\frac{\partial }{\partial r}-\frac{1}{r}\right)v\left(r,t\right), \end{array}$$ (1)
  where *τ*(*r*, *t*) = *T*_*rϕ*_ is the non-zero shear stress, *μ* is the viscosity, *α*_1_ is the first normal material modulus.
2. The modified Fourier law defined by Shercliff [18] for thermoelectric medium is extended using the Taylor series expansion of time fractional order would take the form [38]
  $$\begin{array}{c} \left(1+\frac{\tau_{0}^{\alpha }}{\alpha !}D_{t}^{\alpha }\right)q=-\kappa \nabla T+\pi_{0}J, \end{array}$$ (2)
  where *κ* is thermal conductivity, *τ*_0_ is thermal relaxation time, ***q*** is heat conduction vector, *T* is the temperature and ***J*** is the current density vector given by the modified Ohm’s law as follows;
  $$\begin{array}{c} J=\sigma (E+V\times B-k_{0}\nabla T) \end{array}$$ (3)
  *π*_0_ is the Peltier coefficient, *k*_0_ is the Seebeck coefficient at reference temperature *T*_0_ and *σ* is electrical conductivity. The electric intensity vector and the magnetic induction vector are ***E***, ***B*** respectively, where ***B*** has one constant non-vanishing component *B*_*z*_ which has the form;
  $$\begin{array}{c} B_{z}=\mu_{0}H_{0}=B_{0} \end{array}$$ (4)
3. The Lorentz force ***F*** = ***J*** × ***B***, according to the above equations, has one component in *ϕ*-direction of the form:
  $$\begin{array}{c} F_{\phi }=-\sigma B_{0}^{2}v+\sigma k_{0}B_{0}\frac{\partial T}{\partial r} \end{array}$$ (5)

In the above assumptions, $D_{t}^{\alpha },D_{t}^{\beta }$ are Caputo fractional time derivative operators of order *α*, *β* such that 0 < *α*, *β* ≤ 1. Under these assumptions in the absence of polarization voltage, viscous dissipation, as well as heat source, and pressure gradient in the flow direction; the governing equations for such flow will take the form:

The energy equation

$$\begin{array}{c} \rho c_{p}\frac{\partial }{\partial t}\left(1+\frac{\tau_{0}^{\alpha }}{\alpha !}D_{t}^{\alpha }\right)T=\left(\kappa +\sigma \pi_{0}k_{0}\right)\nabla^{2}T-\sigma \pi_{0}B_{0}\left(\frac{1}{r}\frac{\partial }{\partial r}\left(rv\right)\right) \end{array}$$ (6)

where *ρ* is density, *c*_*p*_ is specific heat at constant pressure and ∇^2^ is Laplace operator given by $\nabla^{2}=\frac{\partial^{2}}{\partial r^{2}}+\frac{1}{r}\frac{\partial }{\partial r}$.

The balance of the linear momentum leads to the relevant equation

$$\begin{array}{c} \rho \frac{\partial v(r,t)}{\partial t}=\left(\frac{\partial }{\partial r}+\frac{2}{r}\right)\tau \left(r,t\right)+F_{\phi } \end{array}$$ (7)

Eliminating *τ*(*r*, *t*) between Eqs (1) and (7), we get

$$\begin{array}{c} \frac{\partial v}{\partial t}=\left(\nu +\frac{\alpha_{1}}{\rho }D_{t}^{\beta }\right)\left(\frac{\partial^{2}}{\partial r^{2}}+\frac{1}{r}\frac{\partial }{\partial r}-\frac{1}{r^{2}}\right)v-\frac{\sigma B_{0}^{2}}{\rho }v+\frac{\sigma k_{0}B_{0}}{\rho }\frac{\partial T}{\partial r} \end{array}$$ (8)

The boundary conditions are written as:

$$\begin{array}{cccc} T(r,t) & = & T_{1}H\left(t\right),v\left(r,t\right)=\frac{\nu }{R_{1}}f\left(t\right) & t>0,r=R_{1}\\ T(r,t) & = & T_{2}H\left(t\right),v\left(r,t\right)=\frac{\nu }{R_{1}}g\left(t\right) & t>0,r=R_{2} \end{array}$$ (9)

where *H*(*t*) is the Heaviside unit step function.

Let us introduce dimensionless variables.

$$\begin{array}{ccc} v^{*} & = & \frac{R_{1}}{\nu }v,r^{*}=\frac{r}{R_{1}},t^{*}=\frac{\nu }{R_{1}^{2}}t,\tau_{0}^{*}=\frac{\nu }{R_{1}^{2}}\tau_{0},\theta =\frac{T-T_{0}}{T_{0}}\\ & & \alpha_{1}^{*}={\left(\frac{R_{1}^{2}}{\nu }\right)}^{1-\beta }\alpha_{1},J^{*}=\frac{R_{1}}{\nu \sigma B_{0}}J,\tau^{*}=\frac{R_{1}^{2}}{\nu \mu }\tau \end{array}$$ (10)

where $\nu =\frac{\mu }{\rho }$ is the kinematic viscosity.

Therefore, Eqs (6) and (8) are reduced to the non-dimensional forms (dropping the asterisk for convenience)

$$\begin{array}{c} P_{r}\frac{\partial }{\partial t}\left(1+\frac{\tau_{0}^{\alpha }}{\alpha !}D_{t}^{\alpha }\right)\theta =\left(1+ZT_{0}\right)\nabla^{2}\theta -\Pi_{0}\left(\frac{1}{r}\frac{\partial }{\partial r}\left(rv\right)\right) \end{array}$$ (11)

$$\begin{array}{c} \frac{\partial v}{\partial t}=\left(1+\eta D_{t}^{\beta }\right)\left(\nabla^{2}-\frac{1}{r^{2}}\right)v-M^{2}v+K_{0}\frac{\partial \theta }{\partial r} \end{array}$$ (12)

where; $P_{r}=\frac{c_{p}\mu }{\kappa },M^{2}=\frac{\sigma B_{0}^{2}R_{1}^{2}}{\mu },\eta =\frac{\alpha_{1}}{\rho R_{1}^{2}},K_{0}=\frac{\sigma k_{0}B_{0}T_{0}R_{1}^{2}}{\mu \nu }$, $\Pi_{0}=\frac{\pi_{0}\nu \sigma B_{0}}{\kappa T_{0}},ZT_{0}=\frac{k_{0}^{2}\sigma }{\kappa }T_{0}$

*P*_*r*_, *M*^2^, *η* are Prandtl number, Hartmann number and Viscoelastic parameter and *ZT*_0_ is thermoelectric figure-of-merit [19]

Hence, the boundary condition in non-dimensional form will be

$$\begin{array}{cccc} \theta (r,t) & = & c_{1}H\left(t\right),v\left(r,t\right)=f\left(t\right) & t>0,r=1\\ \theta (r,t) & = & c_{2}H\left(t\right),v\left(r,t\right)=g\left(t\right) & t>0,r=\ell \end{array}$$ (13)

where $c_{1}=\frac{T_{1}}{T_{0}},c_{2}=\frac{T_{2}}{T_{0}}$ and $\ell =\frac{R_{2}}{R_{1}}>1$.

Now consider the potential function Φ which is defined by [42]

$$\begin{array}{c} v=\frac{\partial \Phi }{\partial r} \end{array}$$ (14)

Substitute from Eq (14) into Eqs (11) and (12) and use the relation $\left(\nabla^{2}-\frac{1}{r^{2}}\right)\frac{\partial f}{\partial r}=\frac{\partial }{\partial r}\nabla^{2}f\left(r\right)$, we get

$$\begin{array}{c} P_{r}\frac{\partial }{\partial t}\left(1+\frac{\tau_{0}^{\alpha }}{\alpha !}D_{t}^{\alpha }\right)\theta =\left(1+ZT_{0}\right)\nabla^{2}\theta -\Pi_{0}\nabla^{2}\Phi \end{array}$$ (15)

$$\begin{array}{c} \frac{\partial \Phi }{\partial t}=\left(1+\eta D_{t}^{\beta }\right)\nabla^{2}\Phi -M^{2}\Phi +K_{0}\theta \end{array}$$ (16)

Applying the Laplace transform with parameter *s* for both sides of Eqs (15) and (16) and using the homogenous initial condition [43], we obtain

$$\begin{array}{c} P_{r}s\left(1+\frac{\tau_{0}^{\alpha }}{\alpha !}s^{\alpha }\right)\bar{\theta }=\left(1+ZT_{0}\right)\nabla^{2}\bar{\theta }-\Pi_{0}\nabla^{2}\bar{\Phi } \end{array}$$ (17)

$$\begin{array}{c} \left(s+M^{2}\right)\bar{\Phi }=\left(1+\eta s^{\beta }\right)\nabla^{2}\bar{\Phi }+K_{0}\bar{\theta } \end{array}$$ (18)

The boundary conditions take the form

$$\begin{array}{cccc} \bar{\theta }\left(r,s\right) & = & \frac{c_{1}}{s},\frac{\partial \bar{\Phi }}{\partial r}=F\left(s\right) & r=1\\ \bar{\theta }\left(r,s\right) & = & \frac{c_{2}}{s},\frac{\partial \bar{\Phi }}{\partial r}=G\left(s\right) & r=\ell \end{array}$$ (19)

Now, we can rewrite Eqs (17) and (18) in the form

$$\begin{array}{c} \left(\nabla^{2}-a_{1}\right)\bar{\theta }=a_{2}\nabla^{2}\bar{\Phi } \end{array}$$ (20)

$$\begin{array}{c} \left(\nabla^{2}-b_{1}\right)\bar{\Phi }=-b_{2}\bar{\theta } \end{array}$$ (21)

where $a_{1}=\frac{p_{r}s}{1+ZT_{0}}\left(1+\frac{\tau_{0}^{\alpha }}{\alpha !}s^{\alpha }\right),a_{2}=\frac{\Pi_{0}}{1+ZT_{0}},b_{1}=\frac{s+M^{2}}{1+\eta s^{\beta }}$ and $b_{2}=\frac{K_{0}}{1+\eta s^{\beta }}$

Eliminating $\bar{\theta }$ between Eqs (20) and (21), we obtain the following fourth order differential equation satisfied by $\bar{\Phi }$

$$\begin{array}{c} \left[\nabla^{4}-\left(a_{1}+b_{1}-a_{2}b_{2}\right)\nabla^{2}+a_{1}b_{1}\right]\bar{\Phi }=0 \end{array}$$ (22)

Eq (22) can be factorized as

$$\begin{array}{c} \left(\nabla^{2}-k_{1}^{2}\right)\left(\nabla^{2}-k_{2}^{2}\right)\bar{\Phi }=0 \end{array}$$ (23)

where both $k_{1}^{2}$ and $k_{2}^{2}$ are the roots of the characteristic equation

$$\begin{array}{c} k^{4}-\left(a_{1}+b_{1}-a_{2}b_{2}\right)k^{2}+a_{1}b_{1}=0 \end{array}$$ (24)

The solution of Eq (23) can be written as

$$\begin{array}{c} \bar{\Phi }=\sum_{i=1}^{2}{\bar{\Phi }}_{i} \end{array}$$ (25)

where ${\bar{\Phi }}_{i}$ is the solution of equation

$$\begin{array}{c} \left(\nabla^{2}-k_{i}^{2}\right)\bar{\Phi }=0,i=1,2 \end{array}$$ (26)

Thus the general solution of Eq (23) has the form

$$\begin{array}{c} \bar{\Phi }=\sum_{i=1}^{2}\left[A_{i}\left(s\right)I_{0}\left(k_{i}r\right)+B_{i}\left(s\right)K_{0}\left(k_{i}r\right)\right] \end{array}$$ (27)

where *A*_*i*_(*s*) and *B*_*i*_(*s*), *i* = 1, 2 are parameters to be determined from the boundary conditions while *I*_0_(*k*_*i*_*r*) and *K*_0_(*k*_*i*_*r*) are the modified Bessel functions of the first and second kinds, respectively. By the same manner, we get

$$\begin{array}{c} \bar{\theta }=\sum_{i=1}^{2}\left[C_{i}\left(s\right)I_{0}\left(k_{i}r\right)+N_{i}\left(s\right)K_{0}\left(k_{i}r\right)\right] \end{array}$$ (28)

where *C*_*i*_(*s*) and *N*_*i*_(*s*), *i* = 1, 2 are parameters to be determined from the boundary conditions. The compatibility between Eqs (27), (28) and (21) gives

$$\begin{array}{c} C_{i}=-\frac{k_{i}^{2}-b_{1}}{b_{2}}A_{i} \end{array}$$ (29)

$$\begin{array}{c} N_{i}=-\frac{k_{i}^{2}-b_{1}}{b_{2}}B_{i} \end{array}$$ (30)

Substituting from Eqs (29) and (30) into Eq (28), we get

$$\begin{array}{c} \bar{\theta }=-\frac{1}{b_{2}}\sum_{i=1}^{2}\left[A_{i}\left(k_{i}^{2}-b_{1}\right)I_{0}\left(k_{i}r\right)+B_{i}\left(k_{i}^{2}-b_{1}\right)K_{0}\left(k_{i}r\right)\right] \end{array}$$ (31)

Using Eq (27) to determine the velocity, Eq (14), will take the form

$$\begin{array}{c} \bar{v}=\sum_{i=1}^{2}\left[A_{i}k_{i}I_{1}\left(k_{i}r\right)-B_{i}k_{i}K_{1}\left(k_{i}r\right)\right] \end{array}$$ (32)

Applying the Laplace transform for the non-dimensional form of Eq (1), we get

$$\begin{array}{c} \bar{\tau }=\left(1+\eta s^{\beta }\right)\left(\frac{\partial }{\partial r}-\frac{1}{r}\right)\bar{v} \end{array}$$ (33)

Using Eq (32) we obtain

$$\begin{array}{c} \bar{\tau }=\left(1+\eta s^{\beta }\right)\sum_{i=1}^{2}\left\{A_{i}\left[k_{i}^{2}I_{0}\left(k_{i}r\right)-\frac{2k_{i}}{r}I_{1}\left(k_{i}r\right)\right]+B_{i}\left[k_{i}^{2}K_{0}\left(k_{i}r\right)+\frac{2k_{i}}{r}K_{1}\left(k_{i}r\right)\right]\right\} \end{array}$$ (34)

The Laplace transform of the non-dimensional form of Eq (3) is given by

$$\begin{array}{c} \bar{J}=\bar{v}-K_{c}\frac{\partial \bar{\theta }}{\partial r} \end{array}$$ (35)

After substituting from Eqs (31) and (32) into Eq (35) and doing some manipulations, we obtain

$$\begin{array}{c} \bar{J}=\sum_{i=1}^{2}\left\{k_{i}\left(1+\frac{K_{c}}{b_{2}}\left(k_{i}^{2}-b_{1}\right)\right)\left[A_{i}I_{1}\left(k_{i}r\right)-B_{i}K_{1}\left(k_{i}r\right)\right]\right\} \end{array}$$ (36)

where $K_{c}=\frac{k_{0}T_{0}}{\nu B_{0}}$

Using the boundary conditions Eq (19), we get

$$\begin{array}{c} -\frac{1}{b_{2}}\sum_{i=1}^{2}\left[A_{i}\left(k_{i}^{2}-b_{1}\right)I_{0}\left(k_{i}\right)+B_{i}\left(k_{i}^{2}-b_{1}\right)K_{0}\left(k_{i}\right)\right]=\frac{c_{1}}{s} \end{array}$$ (37)

$$\begin{array}{c} -\frac{1}{b_{2}}\sum_{i=1}^{2}\left[A_{i}\left(k_{i}^{2}-b_{1}\right)I_{0}\left(k_{i}\ell \right)+B_{i}\left(k_{i}^{2}-b_{1}\right)K_{0}\left(k_{i}\ell \right)\right]=\frac{c_{2}}{s} \end{array}$$ (38)

$$\begin{array}{c} \sum_{i=1}^{2}\left[A_{i}k_{i}I_{1}\left(k_{i}\right)-B_{i}k_{i}K_{1}\left(k_{i}\right)\right]=F\left(s\right) \end{array}$$ (39)

$$\begin{array}{c} \sum_{i=1}^{2}\left[A_{i}k_{i}I_{1}\left(k_{i}\ell \right)-B_{i}k_{i}K_{1}\left(k_{i}\ell \right)\right]=G\left(s\right) \end{array}$$ (40)

By solving the above system, the solution of the problem in the transformed domain could be obtained.

## Inversion of the Laplace transforms

In order to invert the Laplace transform, we adopt a numerical inversion method based on a Fourier series expansion. By this method the Laplace inverse of the function $\bar{f}\left(s\right)$ is approximated by [44]

$$\begin{array}{c} f\left(t\right)=\frac{\exp(ct)}{t_{1}}\left[\frac{1}{2}\bar{f}\left(c\right)+Re\sum_{k=1}^{N}\bar{f}\left(c+\frac{ik\pi }{t_{1}}\right)\exp\left(\frac{ik\pi t}{t_{1}}\right)\right],0<t<2t_{1} \end{array}$$

where $i=\sqrt{-}1$, *N* is a sufficiently large integer representing the number of terms in the truncated Fourier series, chosen such that

$$\begin{array}{c} \exp\left(ct\right)Re\left[\bar{f}\left(c+\frac{iN\pi }{t_{1}}\right)\exp\left(\frac{iN\pi t}{t_{1}}\right)\right]\leq \epsilon_{1} \end{array}$$

where *ϵ*_1_ is a prescribed small positive number that corresponds to the degree of accuracy required. The parameter c is a positive free parameter that must be greater than the real part of all the singularities of $\bar{f}\left(c\right)$. The optimal choice of c was obtained according to the criteria described in [44].

## Numerical results and discussion

The polymer fluid chosen for purposes of numerical evaluations is 0.2% Xanthan Gum (XG) [45, 46]. XG solution has been used extensively in the oil industry for different applications due to its unique rheological properties. The experimental results indicate that fines generated during the drilling process form an external filter cake which in combination with XG results in considerable fluid loss reduction. The damage due to XG is small and limited to a narrow thickness around the wellbore [47]. The constants of the problem are shown in Table 1.

**Table 1:** The constants of the problem.

*η* = 0.805	*M*^2^ = 0.25	Π_0_ = 0.8	*K*_0_ = 2
*τ*_0_ = 0.015	*P*_*r*_ = 0.71	*K*_*c*_ = 8	*ZT*_0_ = 1
*c*_1_ = 1	*c*_2_ = 1	*ℓ* = 2	

The computations were carried out for chosen functions *f*(*t*) = −*g*(*t*) = *H*(*t*). The temperature, the velocity, the stress, and current density are calculated numerically using the inversion of Laplace transform outlined above. The FORTRAN programming language is used to solve the problem. The accuracy maintained is 6 digits in the numerical program.

**Fig 2:**
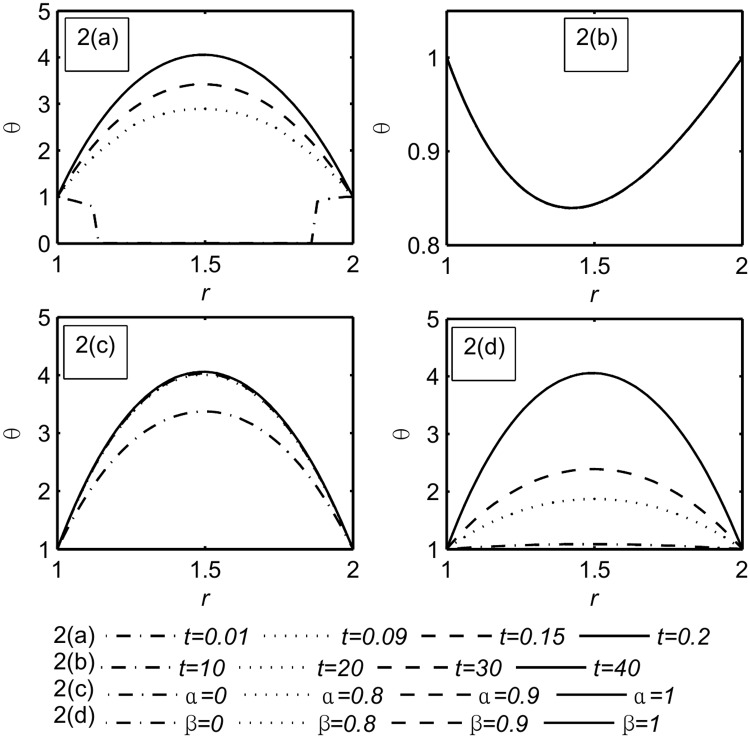
Temperature distribution *θ*. Part(a): For different values of small time, namely, *t* = 0.01, 0.09, 0.15, 0.2 when *α* = *β* = 1. Part(b): For different values of large time, namely, *t* = 10, 20, 30, 40 when *α* = *β* = 1. Part(c): For different values of *α*, namely, *α* = 0, 0.8, 0.9, 1 when *t* = 0.2, *β* = 1. Part(d): For different values of *β*, namely, *β* = 0, 0.8, 0.9, 1 when *t* = 0.2, *α* = 1.

**Fig 3:**
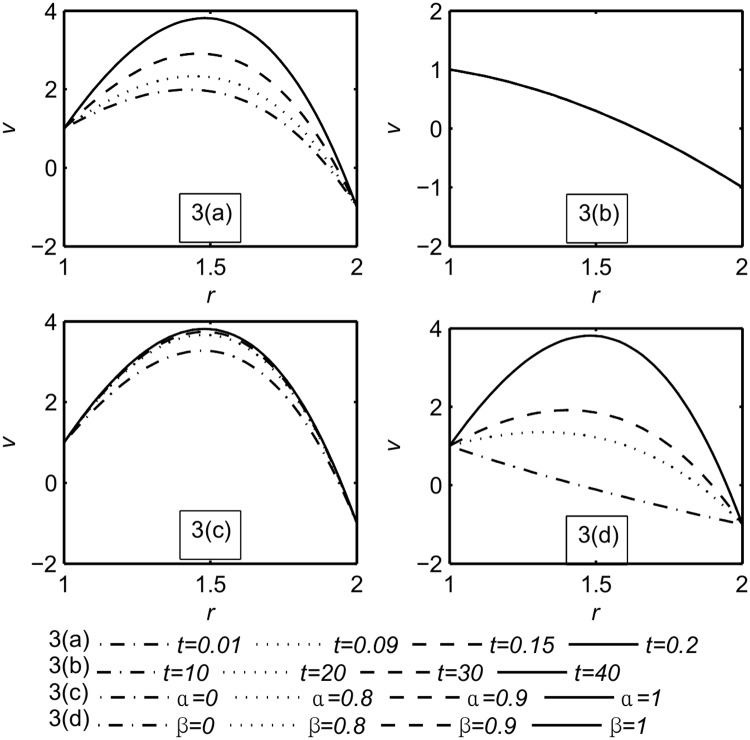
Velocity distribution *v*. Part(a): For different values of small time, namely, *t* = 0.01, 0.09, 0.15, 0.2 when *α* = *β* = 1. Part(b): For different values of large time, namely, *t* = 10, 20, 30, 40 when *α* = *β* = 1. Part(c): For different values of *α*, namely, *α* = 0, 0.8, 0.9, 1 when *t* = 0.2, *β* = 1. Part(d): For different values of *β*, namely, *β* = 0, 0.8, 0.9, 1 when *t* = 0.2, *α* = 1.

**Fig 4:**
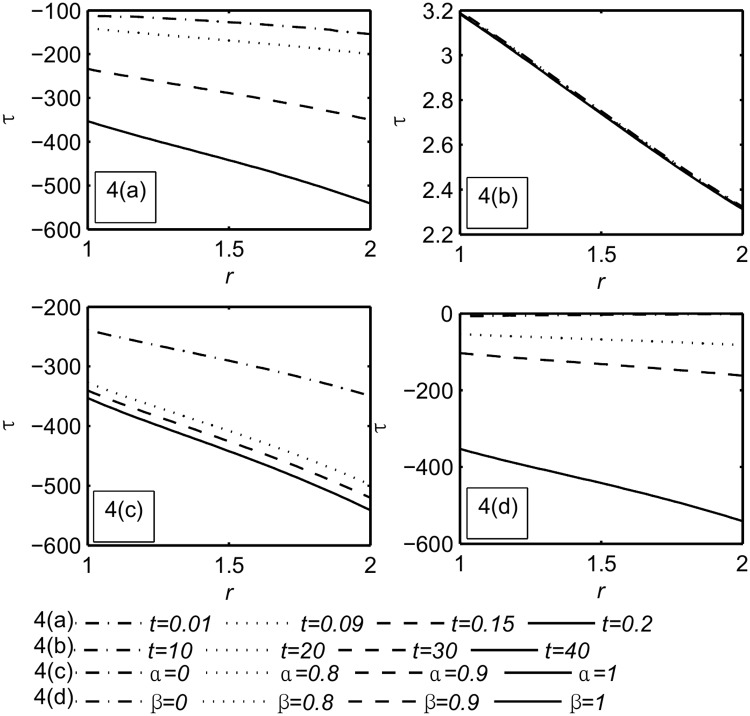
The stress distribution *τ*. Part(a): For different values of small time, namely, *t* = 0.01, 0.09, 0.15, 0.2 when *α* = *β* = 1. Part(b): For different values of large time, namely, *t* = 10, 20, 30, 40 when *α* = *β* = 1. Part(c): For different values of *α*, namely, *α* = 0, 0.8, 0.9, 1 when *t* = 0.2, *β* = 1. Part(d): For different values of *β*, namely, *β* = 0, 0.8, 0.9, 1 when *t* = 0.2, *α* = 1.

Figs (2–4) represent the variables field distributions namely, the temperature, the velocity and the stress, respectively. Each Figure split into four subfigures, part(a) describe the variation of the functions field with small times, namely, *t* = 0.01, 0.09, 0.15, 0.2 when *α* = *β* = 1 while in part(b) the calculation is done for large values of time, namely, *t* = 10, 20, 30, 40 when *α* = *β* = 1. Part(c), (d) illustrate the influence of the fractional parameters *α*, *β* respectively when *t* = 0.2.

We notice that the magnitude of temperature, velocity and stress were increased with the increasing time [30]. Also from Fig 2 Part(a) for the smallest value of time, the generalized theory with one relaxation time (G- theory) is prominent. This is in agreement with [48].

The rotation of the two cylinders divides the fluid region into two parts according to the direction of rotation. The first one is denoted by *R*_*in*_ in which the fluid rotates in the same direction as the inner cylinder while the second part is *R*_*out*_ due to the outer cylinder rotation. It is observed from Fig 3 Part(a) that as the time increases, the value of *R*_*in*_ and the peak of velocity increases, as shown in Table 2.

**Table 2:** The influence of the inner region *R*_*in*_ and peak on the velocity at *α* = *β* = 1 for different time *t*.

*t*	*R*_*in*_	peak
0.01	1.898990	(1.424242, 1.988389)
0.09	1.909091	(1.444444, 2.331563)
0.15	1.929293	(1.464646, 2.907461)
0.2	1.939394	(1.484848, 3.809927)

From Figs (2–4) part(b) it can be noticed that the functions field unchanging at large time *t* = 10, 20, 30, 40. This means that the steady state is achieved. For the steady state analysis, see S1 Text.

Figs (2–4) part(c) it is noticed that the magnitude of the fields distribution increases with the increase of *α*. Fig 3 Part(c) shows the *α* influence on *R*_*in*_ is insignificant while the peak of the velocity increases when *α* increases as shown in Table 3.

**Table 3:** The peak on the velocity at *β* = 1 and *t* = 0.2 for different fractional parameter *α*.

*α*	peak
0.0	(1.474747, 3.270884)
0.8	(1.474747, 3.665751)
0.9	(1.474747, 3.737811)
1	(1.474747, 3.809938)

Figs (2–4) part(d) it is noticed that the magnitude of the temperature, the velocity, and the stress increase with the increasing of *β*. Fig 3 Part(d) shows the *β* influence on *R*_*in*_ and the peak are prominent but for viscous fluid (*β* = 0) the peak disappears (see Table 4).

**Table 4:** The influence of the inner region *R*_*in*_ and peak on the velocity at *α* = 1, *t* = 0.2 for different fractional parameter *β*.

*β*	*R*_*in*_	peak
0.0	1.434343	Non
0.8	1.848485	(1.323232, 1.350466)
0.9	1.88889	(1.404040, 1.911008)
1	1.939394	(1.484848, 3.809927)

From Fig 3 Part(a), (d) it has been observed that for small value of time the influences of the time *t* and the fractional parameter *β* on *R*_*in*_ are prominent. This region extended by increasing both of them until the interaction between *R*_*in*_ and *R*_*out*_ occurs (see S1 Video). Later the inner region returns back towards the inner cylinder (see S2 Video). This is due to the reaction of the fluid particles in the outer region on the inner region. Fig 4 Part(a), (c), (d) show the behavior of the stress distribution at small value of time which is compatible with recent work [30].

**Fig 5:**
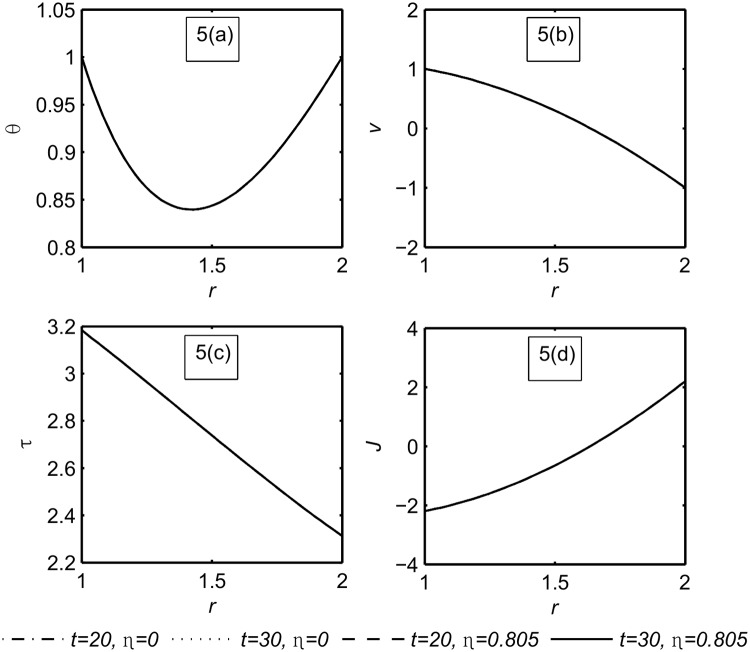
The functions field *θ*, *v*, *τ*, *J* at large time *t* = 20, *t* = 30 for both Newtonian (*η* = 0) and non-Newtonian fluid (*η* = 0.805) when *α* = *β* = 1.

Fig 5(a)–5(d) depicts the variations of the temperature *θ*, the velocity *v*, the stress *τ*, and the current density *J*, respectively, at large values of time (*t* = 20, *t* = 30) for both Newtonian (*η* = 0) and non-Newtonian fluids (*η* = 0.805) at *α* = *β* = 1.

**Fig 6:**
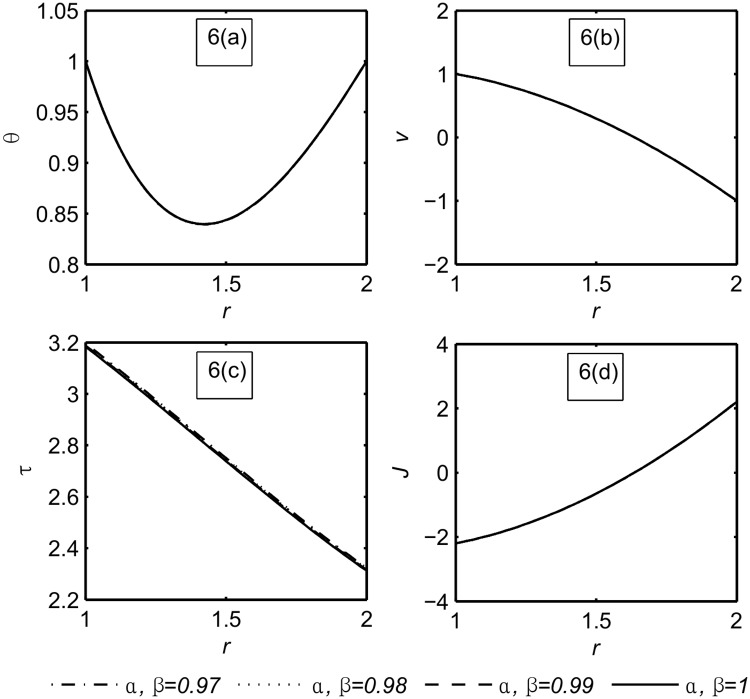
The functions field *θ*, *v*, *τ*, *J* at large time *t* = 20 and different large values of *α*, *β* = 0.97, 0.98, 0.99, 1.

It has been observed that for large times all the functions field are the same for the Newtonian and non-Newtonian fluids. This result is compatible with the physical phenomena that for a large time the steady state is achieved due to the viscosity of the fluid. Now, there is a question arises here; Is the change of the fraction parameters effect on the functions field distribution in the case of large values of time? The answer is obtained from Fig 6(a)–6(d).

This figure shows the functions field for large value of time, *t* = 20 as well as for different large values of *α*, *β*. It is noticed that the fractional parameters have no effect on all functions field. Hence mulling over Figs 5 and 6 we conclude that when steady state occurs the fractional parameters have no effect on all functions field (see S3 Video).

The influences of the physical parameters on all functions field at small and large value of times, namely, *t* = 0.2 and *t* = 20 are shown in S1–S8 Figs.

It is observed from S1 Fig that, for small values of time, the effect of increasing Prandtl number *P*_*r*_ is to decrease the values of the temperature, the velocity and the current. On the other hand the increase Prandtl number *P*_*r*_ tends to increase the value of the stress. The physical explanation of the above observation is that increasing *P*_*r*_ tends to decrease the thermal conductivity of the fluid resulting on a lower temperature. Also, increasing *P*_*r*_ increases the viscosity of the fluid producing higher stress values and slower speed [12]. We note that for large values of time (shown in S2 Fig) the Prandtl number *P*_*r*_ has no effect on all the functions. This result is compatible with the equations of the steady state which are independent of *P*_*r*_.

From S3 Fig we conclude that increasing the thermoelectric parameter *ZT*_0_ results in increasing the values of the velocity while decreasing the temperature and the stress. This is because increasing *ZT*_0_ decreases the thermal conductivity and increases the Seebeck coefficient and electric conductivity. For large values of time (shown in S4 Fig) the effect of *ZT*_0_ on the stress and on the current is most prominent while it has a small effect on the temperature and a very small effect on the velocity. The increase of *ZT*_0_ decreases the values of the velocity while increasing the values of both the temperature and the stress. The effect on the current is not consistent due to the rotation of parts of the fluid in opposite directions.

We see from S5 Fig that the increase of the Hartmann number *M*^2^ results in the increase of the values of the stress and a decrease in the values of both the temperature and the velocity. As before, the rotation of the fluid affects the current in different ways according to the direction of the rotating fluid. S6 Fig shows that for large values of time, the effect of the Hartmann number *M*^2^ on the temperature, the velocity and the stress is the same (with different values) as in S5 Fig. The graph of the current shows that: it increases consistently with the increase of the Hartmann number. S7 and S8 Figs show that for small or large values of time, the parameter *K*_*c*_, as expected from Eq (35), affects the current only and has no effect on the other functions.

## Conclusions

The present study describes the fractional mathematical model of an unsteady rotational flow of Xanthan gum (XG) between two cylinders in the presence of a transverse magnetic field. The cylinders rotate in a counter direction and affected with thermal shock that is function of time.

The summary of this study for different time intervals are as follow:

### For small values of time

- In all related figures, it is noticed that the thermomechanical fractional parameters *α* and *β* have a significant effect on all fields.
- The fractional parameter *β* controls the rotation of the inner region *R*_*in*_ while the fractional parameter *α* has nuance effect on this region.
- The inner rotated region *R*_*in*_ extended by the time, as the fractional parameter *β* increases.
- In the presence of the applied magnetic field, the fluid motion is directly affected by the rotation of the inner cylinder more than the rotation of the outer cylinder.
- It has been found that; this model for small values of time, the temperature profile is in agreement with generalized theory of one relaxation time.

### For large values of time

- Strong non-Newtonian effect is present for small value of time while for large times it becomes weak and behaves like a Newtonian fluid.
- The change of the fractional parameters *α*, *β* has no considerable effect on the functions field.

## Supporting Information

S1 Video 3D simulation of the fractional parameter effects on the two coaxial rotating cylinders for small value of time. This video explains the movement of the inner fluid region *R*_*in*_ where its boundary is represented by a moving blue cylinder for different values of the fractional parameter *β*. It is observed that after a certain duration of time, this region is extended towards the outer cylinder as the fractional parameters increase.
(AVI)

S2 Video 3D simulation of the time effects on the two coaxial rotating cylinders after interaction occurs. After the interaction of the fluid regions occur, the inner flow returns back towards the inner cylinder. This is due to the reaction of the fluid particles in the outer region on the particles in the inner region.
(AVI)

S3 Video 3D simulation of the time effects on the two coaxial rotating cylinders for large value of time. In this case, the steady state occurs and the Non-Newtonian fluid behaves like Newtonian one whatever the value of *α* and *β*.
(AVI)

S1 Text Steady state analysis. This text contains some more detailed information about the functions field at large value of time *t* where the steady state is achieved.
(DOC)

S1 Fig The influence of *P*_*r*_ on all functions field at small value of time. The functions field *θ*, *v*, *τ*, *J* at small time *t* = 0.2 and different values of Prandtl number *P*_*r*_ = 0.5, 0.6, 0.71, 0.8.
(PDF)

S2 Fig The influence of *P*_*r*_ on all functions field at large value of time. The functions field *θ*, *v*, *τ*, *J* at large time *t* = 20 and different values of Prandtl number *P*_*r*_ = 0.5, 0.6, 0.71, 0.8.
(PDF)

S3 Fig The influence of *ZT*_0_ on all functions field at small value of time. The functions field *θ*, *v*, *τ*, *J* at small time *t* = 0.2 and different values of Thermoelectric figure-of-merit *ZT*_0_ = 1, 1.5, 1.7, 2.
(PDF)

S4 Fig The influence of *ZT*_0_ on all functions field at large value of time. The functions field *θ*, *v*, *τ*, *J* at large time *t* = 20 and different values of Thermoelectric figure-of-merit *ZT*_0_ = 1, 1.5, 1.7, 2.
(PDF)

S5 Fig The influence of *M*^2^ on all functions field at small value of time. The functions field *θ*, *v*, *τ*, *J* at small time *t* = 0.2 and different values of Hartmann number *M*^2^ = 0, 0.25, 0.5, 1.
(PDF)

S6 Fig The influence of *M*^2^ on all functions field at large value of time. The functions field *θ*, *v*, *τ*, *J* at large time *t* = 20 and different values of Hartmann number *M*^2^ = 0, 0.25, 0.5, 1.
(PDF)

S7 Fig The influence of *K*_*c*_ on all functions field at small value of time. The functions field *θ*, *v*, *τ*, *J* at small time *t* = 0.2 and different values of *K*_*c*_, namely, *K*_*c*_ = 0.2, 0.8, 2, 8.
(PDF)

S8 Fig The influence of *K*_*c*_ on all functions field at large value of time. The functions field *θ*, *v*, *τ*, *J* at large time *t* = 20 and different values of *K*_*c*_, namely, *K*_*c*_ = 0.2, 0.8, 2, 8.
(PDF)
