# Supplementary material for: Thermomechanical Fractional Model of TEMHD Rotational Flow
Source: PLoS One. 2017 Jan 3;12(1):e0168530. doi: 10.1371/journal.pone.0168530 (PMC5207645; doi:10.1371/journal.pone.0168530)
Supplement: S1 Text — This text contains some more detailed information about the functions field at large value of time t where the steady state is achieved. (DOC) [file pone.0168530.s004.doc]

Steady state analysis:

From Figs.(2 - 4) part (b) it can be noticed that the functions field unchanging at large time. This means that the partial derivative of these functions with respect to time is zero. Under this condition the governing equations have the following forms: $\tau=\mu\left(\frac{d}{dr}-\frac{1}{r}\right) v$ (A.1)

$\text{(}\kappa+\sigma\pi_{o}k_{o}\text{)} \nabla^{2} T-\sigma\pi_{o} B_{o} \left(\frac{1}{r} \frac{d}{dr} \left(r v\right)\right)=0$, (A.2)

$\left(\frac{d}{dr}+\frac{2}{r}\right) \tau\text{(}r\text{,}t\text{)}+\text{ }F_{\phi}=0$ (A.3)

Eliminating $\tau\text{(}r\text{)}$ between Eq. (A.1) and Eq. (A.3), we get

$\upsilon\left(\frac{d^{2}}{dr^{2}}+\frac{1}{r} \frac{d}{dr}-\frac{1}{r^{2}}\right) v-\frac{\sigma B_{o}^{2}}{\rho}v+\frac{\sigma k_{o}B_{o}}{\rho}\frac{dT}{dr}=0$ (A.4)

The non-dimension forms of the above Eqs. (A.2, A.4) will take the forms

$\text{(}1+ZT_{o}\text{)} \nabla^{2} \theta-\Pi_{o} \left(\frac{1}{r} \frac{d}{dr} \left(r v\right)\right)=0$, (A.5)

$\left(\nabla^{2}-\frac{1}{r^{2}}\right) v-M^{2} v+K_{o} \frac{d\theta}{dr}=0$, (A.6)

Using the potential function $\Phi$ defined by $v=\frac{\partial\Phi}{\partial r}$, we get

$\nabla^{2} \theta-\frac{\Pi_{o}}{\text{(}1+ZT_{o}\text{)}} \nabla^{2}\Phi=0$, (A.7)

$\left(\nabla^{2} -M^{2}\right) \Phi+K_{o} \theta=0$. (A.8)

Solving the above equations, we obtain

$\nabla^{2} \left(\nabla^{2}+\lambda^{2}\right) \Phi=0$ (A.9)

where $\lambda^{2}= \frac{K_{o}\Pi_{o}}{\text{(}1+ZT_{o}\text{)}}-M^{2}$.

This equation can be obtained from Eq. (22) as a limited case $s\to0$. This is compatible with the final value theorem (FVT) associated with Laplace.

Thus the general solution of Eq. (A.9) has the form

$\Phi=A_{1} \ln\text{(}r\text{)}+B_{1}+A_{2} J_{0}\text{(}\lambda r\text{)}+B_{2} Y_{0}\text{(}\lambda r\text{)}$ (A.10)

where $J_{0}$and $Y_{0}$ are Bessel functions of the first and second kind of order zero

Using Eq. (A.8), we get

$\theta=\frac{M^{2}}{K_{o}}\left(A_{1} \ln\text{(}r\text{)}+B_{1}\right)+ \left(\frac{\lambda^{2}+M^{2}}{K_{o}}\right) \left(A_{2} J_{0}\text{(}\lambda r\text{)}+ B_{2} Y_{0}\text{(}\lambda r\text{)}\right)$ (A.11)

Using Eq. (A.10) to determine the velocity, we get

$v=\frac{A_{1}}{r}-\lambda\left(A_{2} J_{1}\text{(}\lambda r\text{)}+B_{2} Y_{1}\text{(}\lambda r\text{)}\right)$ (A.12)

where $J_{1}$and $Y_{1}$ are Bessel functions of the first and second kind of order one

The non-dimensional form of Eq. (A.1), will take the form

$\tau\text{(}r\text{)}= \left(\frac{d}{dr}-\frac{1}{r}\right) v$ (A.13)

Using Eq. (A.12), and doing some manipulations, we get

$\tau=-\frac{2A_{1}}{r^{2}}+A_{2} \lambda\text{[}\frac{2 }{r} J_{1}\text{(}\lambda r\text{)} - \lambda J_{0}\text{(}\lambda r\text{) ]}+B_{2} \lambda\text{[}\frac{2 }{r} Y_{1}\text{(}\lambda r\text{)} - \lambda Y_{0}\text{(}\lambda r\text{) ]}$ (A.14)

The non-dimensional boundary conditions take the form

(A.15)

where .

Substitute from Eq. (A.11) and Eq. (A.12) into Eq. (A.15), we get

$\frac{M^{2}}{K_{o}}B_{1}+ \left(\frac{\lambda^{2}+M^{2}}{K_{o}}\right) \left(A_{2} J_{0}\text{(}\lambda\text{)}+ B_{2} Y_{0}\text{(}\lambda\text{)}\right)=1$ (A.16)

(A.17)

$A_{1}-\lambda\left(A_{2} J_{1}\text{(}\lambda\text{)}+B_{2} Y_{1}\text{(}\lambda\text{)}\right)=1$ (A.18)

(A.19)

By solving the above system, the exact solution of the problem in the steady state is obtained.
