# Supplementary figures and images for: Thermomechanical Fractional Model of TEMHD Rotational Flow
Source: PLoS One. 2017 Jan 3;12(1):e0168530. doi: 10.1371/journal.pone.0168530 (PMC5207645; doi:10.1371/journal.pone.0168530)

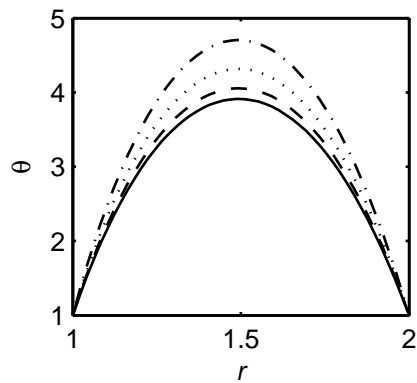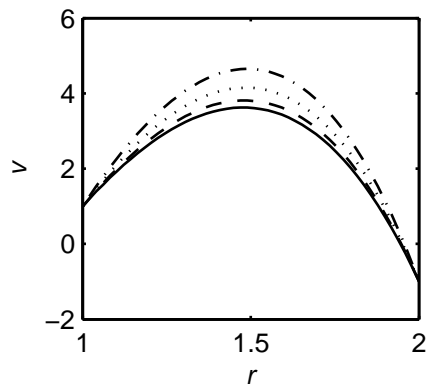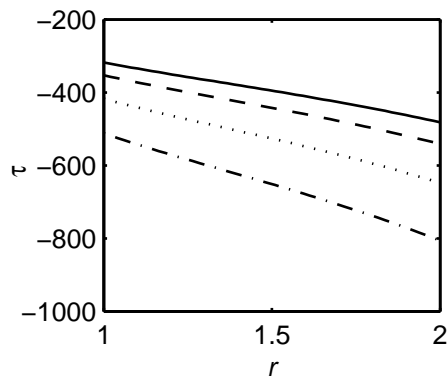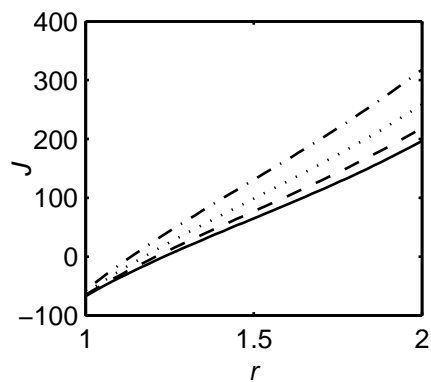

$t=0.2$ ;  $\cdots P_r=0.5$   $\cdots P_r=0.6$   $- - - P_r=0.71$   $—— P_r=0.8$

Supplement: S1 Fig — The functions field θ, v, τ, J at small time t = 0.2 and different values of Prandtl number Pr = 0.5, 0.6, 0.71, 0.8. (PDF) [file pone.0168530.s005.pdf]

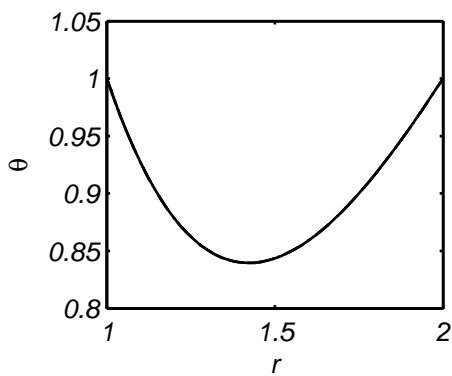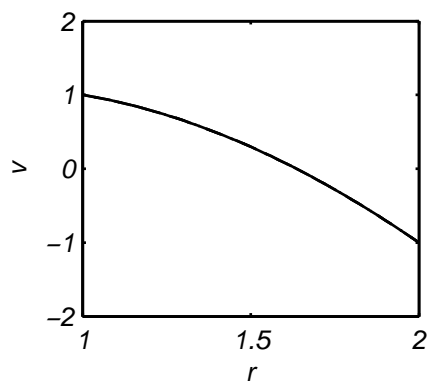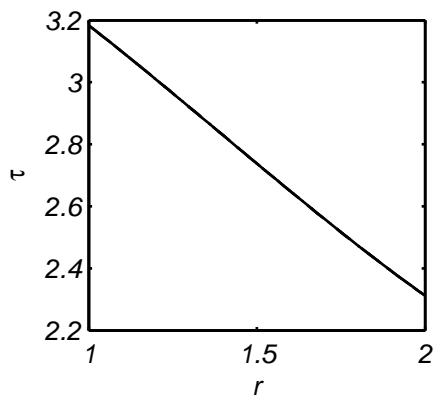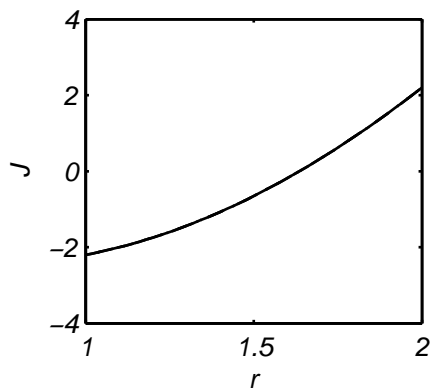

$t=20$ ;  $\cdots \cdots P_r=0.5$   $\cdots \cdots P_r=0.6$   $- - - P_r=0.71$   $—— P_r=0.8$

Supplement: S2 Fig — The functions field θ, v, τ, J at large time t = 20 and different values of Prandtl number Pr = 0.5, 0.6, 0.71, 0.8. (PDF) [file pone.0168530.s006.pdf]

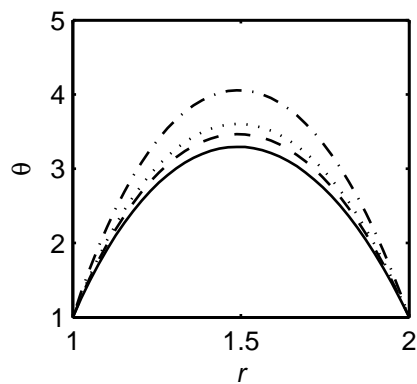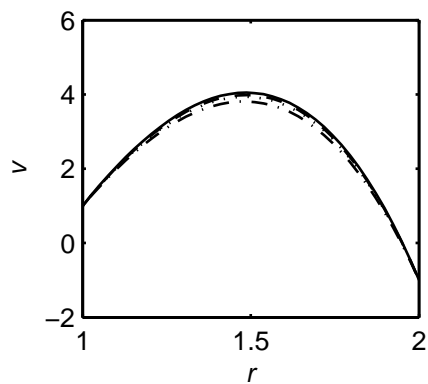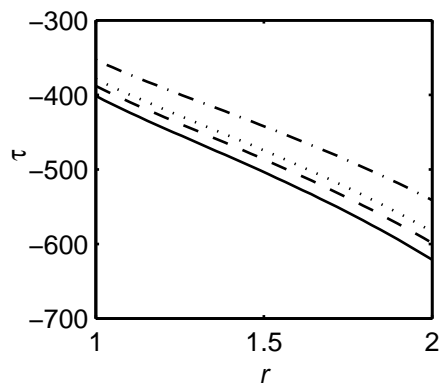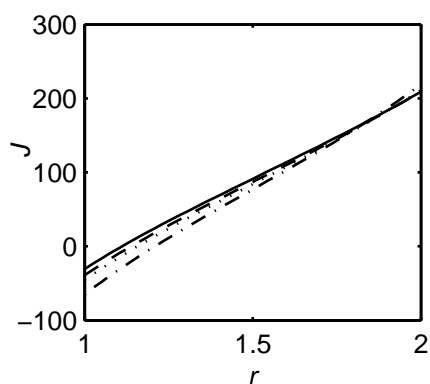

$t=0.2$ ;  $-\cdot-\cdot-$   $ZT_0=1$   $\cdots$   $ZT_0=1.5$   $-\cdot-\cdot-$   $ZT_0=1.7$   $—$   $ZT_0=2$

Supplement: S3 Fig — The functions field θ, v, τ, J at small time t = 0.2 and different values of Thermoelectric figure-of-merit ZT0 = 1, 1.5, 1.7, 2. (PDF) [file pone.0168530.s007.pdf]

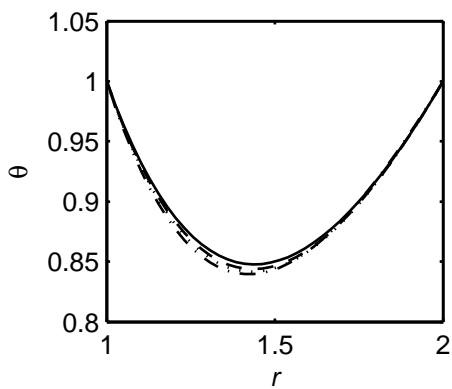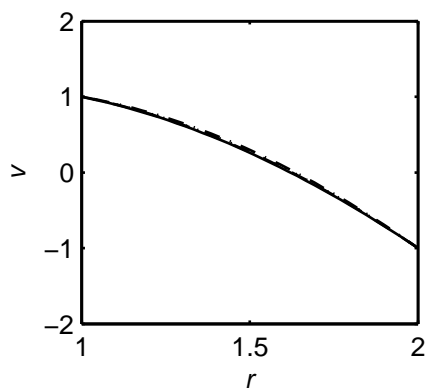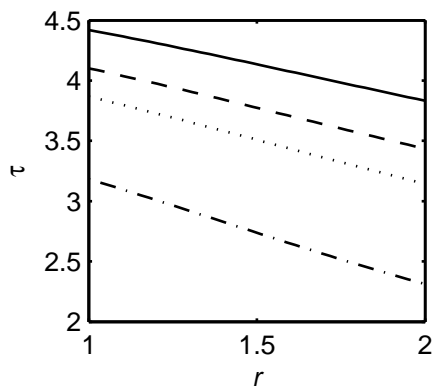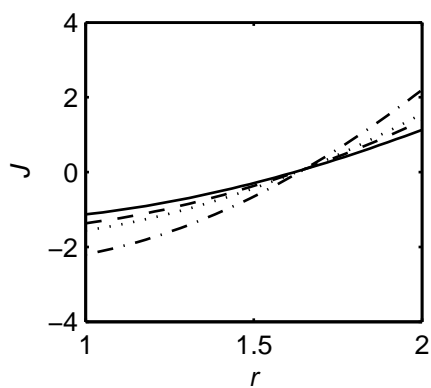

$t=20$ ;  $\cdots\cdots\cdots ZT_0=1$   $\cdots\cdots\cdots ZT_0=1.5$   $-\cdots-\cdots ZT_0=1.7$   $\text{——} ZT_0=2$

Supplement: S4 Fig — The functions field θ, v, τ, J at large time t = 20 and different values of Thermoelectric figure-of-merit ZT0 = 1, 1.5, 1.7, 2. (PDF) [file pone.0168530.s008.pdf]

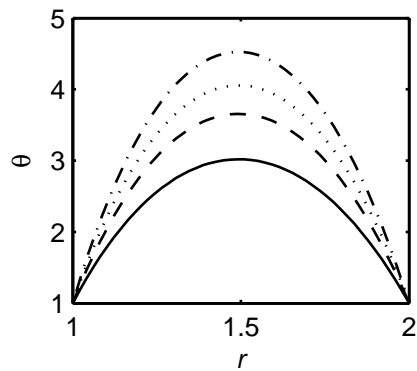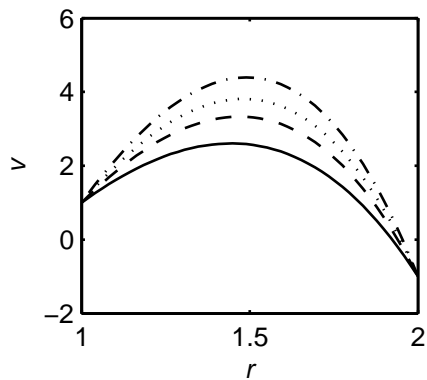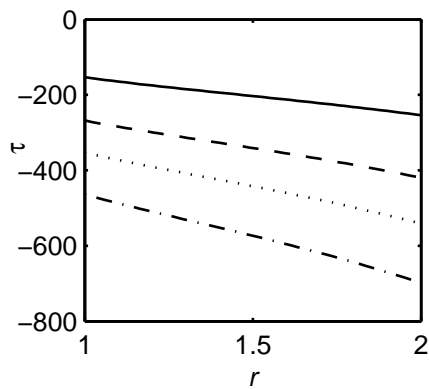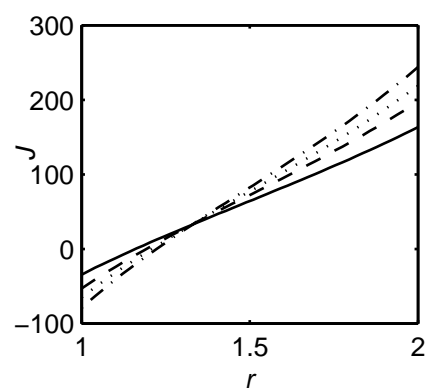

$t=0.2$ ;  $\cdots$   $M^2=0$   $\cdots$   $M^2=0.25$   $- - -$   $M^2=0.5$   $—$   $M^2=1$

Supplement: S5 Fig — The functions field θ, v, τ, J at small time t = 0.2 and different values of Hartmann number M2 = 0, 0.25, 0.5, 1. (PDF) [file pone.0168530.s009.pdf]

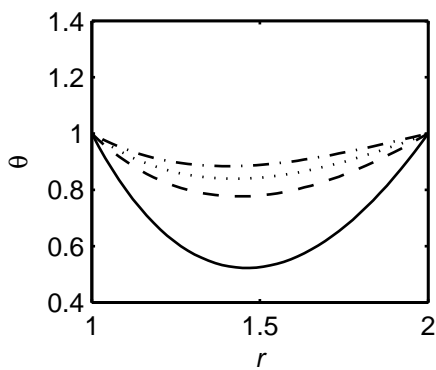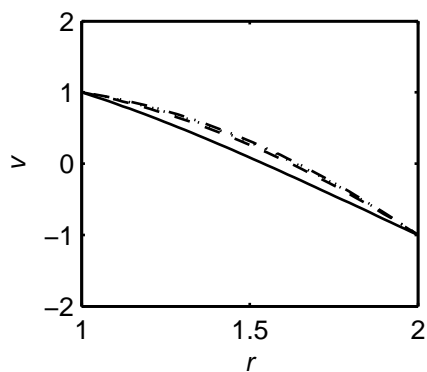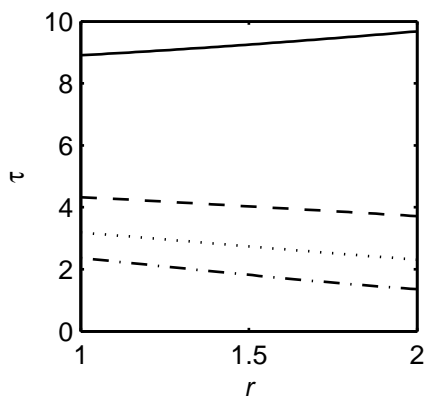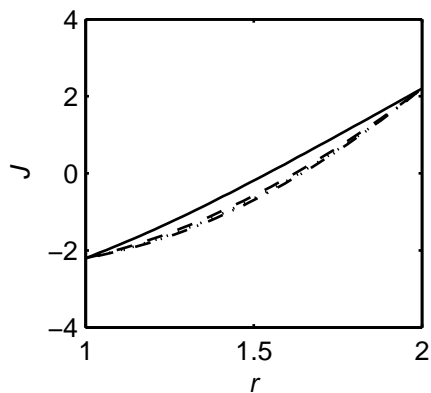

$t=20$ ;  $\cdots \cdots M^2=0$   $\cdots \cdots M^2=0.25$   $- - - M^2=0.5$   $—— M^2=1$

Supplement: S6 Fig — The functions field θ, v, τ, J at large time t = 20 and different values of Hartmann number M2 = 0, 0.25, 0.5, 1. (PDF) [file pone.0168530.s010.pdf]

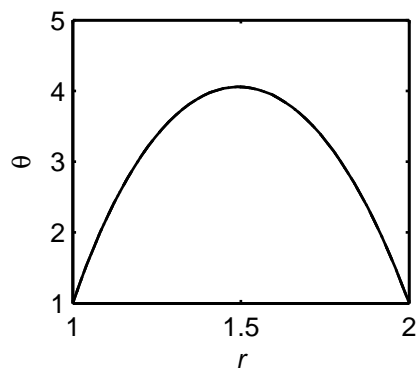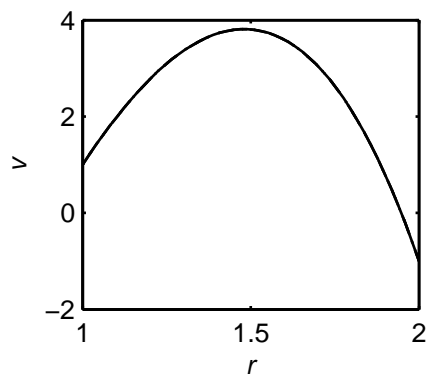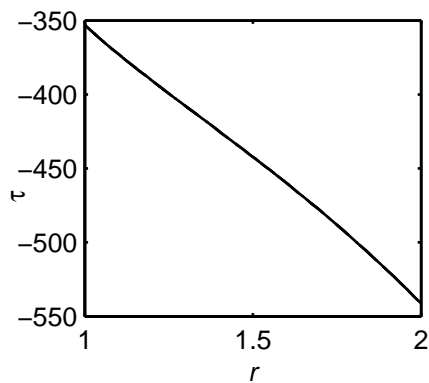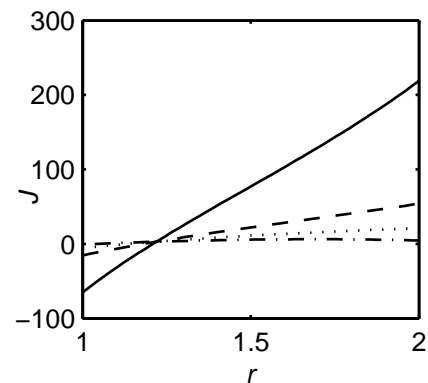

$t=0.2$ ; . - . - .  $K_c=0.2$     . . . . .  $K_c=0.8$     - - -  $K_c=2$     —  $K_c=8$

Supplement: S7 Fig — The functions field θ, v, τ, J at small time t = 0.2 and different values of Kc, namely, Kc = 0.2, 0.8, 2, 8. (PDF) [file pone.0168530.s011.pdf]

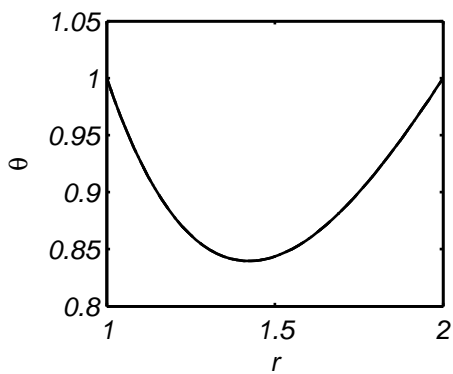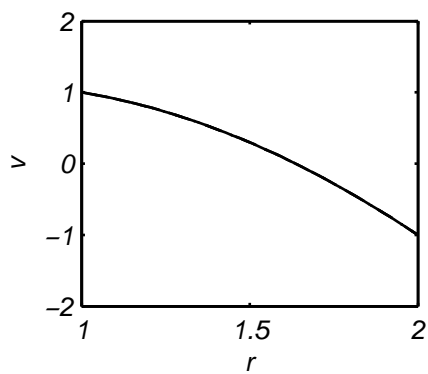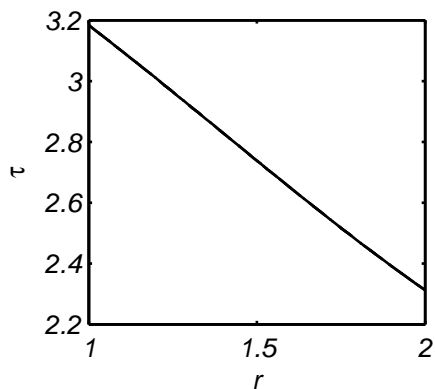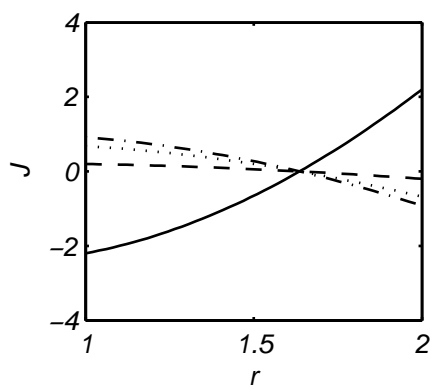

$t=20$ ; - - -  $K_c=0.2$  .....  $K_c=0.8$  - - -  $K_c=2$  ———  $K_c=8$

Supplement: S8 Fig — The functions field θ, v, τ, J at large time t = 20 and different values of Kc, namely, Kc = 0.2, 0.8, 2, 8. (PDF) [file pone.0168530.s012.pdf]
